# Dietary Patterns of Children with Autism Spectrum Disorder: A Study Based in Egypt

**DOI:** 10.3889/oamjms.2015.051

**Published:** 2015-05-07

**Authors:** Nagwa Meguid, Mona Anwar, Safaa Zaki, Wafaa Kandeel, Nihad Ahmed, Ihab Tewfik

**Affiliations:** 1 *National Research Centre, Research on Children with Special Needs Department, Giza, Egypt*; 2 *National Research Centre, Child Health Department, Giza, Egypt*; 3 *National Research Centre, Biological Anthropology Department, Giza, Egypt*; 4 *National Research Centre, Food Science and Nutrition Department, Giza, Egypt*; 5 *Faculty of Science and Technology, University of Westminster, Life Science Department, London, United Kingdom*

**Keywords:** Autistic Children, anthropometric measurements, dietary intake, micronutrients, Egyptian

## Abstract

**AIM:**

In the hope to assist in tailoring individualized nutritional therapy, this study aimed to assess the nutritional status of autistic children.

**MATERIAL AND METHODS:**

This cross-sectional study included 80 autistic children, divided into two groups: group 1 (aged 3- 5 years) and group 2 (aged 6-9 years). Diagnosis was performed based on the criteria for autistic disorder as defined in the Diagnostic and Statistical Manual of Mental Disorders, Fourth Edition, Autism Diagnostic Interview Revised and Autism Rating Scale.

**RESULTS:**

Socio-demographic data, anthropometric measurements and dietary intake patterns were recorded using a validated questionnaire. The daily intakes of calories and nutrients were converted to percentages of the Recommended Dietary Allowance or Dietary Reference Intake based on age- and gender-normalized DRIs. Plotting on the Egyptian sex-specific growth chart, BMI-z scores of both age groups were slightly overweight. Autistic children suffered inadequate intake of some micronutrients such as vitamin D and C, calcium, folate, magnesium, phosphorus, zinc, and iron, some deficiencies were highly significant especially at older age.

**CONCLUSIONS:**

Tailoring a specially designed balanced diet with appropriate micronutrient supplementation may ameliorate the severity of autism symptoms and related abnormal behaviours.

## Introduction

Autism Spectrum Disorder (ASD) is a serious problem and present particular challenges for any discipline of medicine. Since the presenting symptoms can be diverse, it engenders difficulty when assigning a specific diagnostic category for these patients. It is well documented that the prevalence of ASD is increasing and is currently estimated to affect 1 in 150 children [1]. Moreover, exponential increases of this sort suggest a strong environmental component to the etiology of the disease. Epidemiological approach has often resulted in contradictory scientific conclusions when its practitioners do not consider the dietary factors that interact and modulate the molecular and genetic mechanisms underlying human metabolism and brain function [2]. This has been the case despite the existence of literature from the field of “nutrigenomics”, which has specifically studied the effects of food and food ingredients on gene expression [3].

Frequent nutritional screening and assessment of children with ASD is an important clinical consideration as they may have multiple risk factors that could amplify the prevalence of nutrient deficiencies. Those children often exhibit nutrition-related medical issues including gastrointestinal discomfort, bowel inflammation, diarrhea, constipation, and acid reflux [4].

Repetitive behaviors and restricted interests, are core feature of autism, and may play a role in dietary selectivity. Children with ASD often resist novel experiences, which may include tasting new foods. In addition, many children with ASD have sensory hypersensitivities and may reject foods due to an aversion to texture, temperature or other characteristics of the foods [5]. Recently attention has focused on the relationship between metabolic, nutritional disturbances and developmental disorders as ASD [6]. Therefore, targeted, individualized nutritional therapy is crucial to managing the complexity of patients with chronic persistent problems like autism as it may influence the severity, presentation, or dynamics of disease [7].

Nevertheless, accurate understanding of the unique nutritional risk of children with ASD is important to clinicians who are responsible for nutritional surveillance and to parents who are concerned about the effects of limited or restricted diets. Therefore the purpose of this study was to investigate and characterize the nutritional status of children with ASD among a group of Egyptian autistic children. This may help to target, individualized nutritional therapy to reduce the symptoms and co morbidities that are associated with autism.

## Material and Methods

This was a cross-sectional study conducted and funded by the National Research Centre (NRC), Egypt. Eighty autistic children attended the autism clinic, NRC, Cairo were recruited. Participants included 41 boys and 39 girls and their ages vary from three to nine years old, they divided into two groups: group 1, aged 3- 5 years and group 2, aged 6-9 years. The diagnosis of ASD was performed based on the criteria for autistic disorder as defined in the Diagnostic and Statistical Manual of Mental Disorders, Fourth Edition (DSM), Autism Diagnostic Interview Revised (ADI-R) and Autism Rating Scale (CARS). Exclusion criteria for participation in the study included: severe sensory problems (visual impairment or hearing loss), significant motor impairments (failure to sit by 12 months, or walk by 24 months), or identified metabolic, genetic, or progressive neurological disorders, based on screening by clinical staff or the use of nutrient supplements. The Research Ethics Committee of National Research Centre approved the study. The consent forms were endorsed by parents and/or caregivers of participating child.

### Assessment of the nutritional status

Parents and/or caregivers of each participant were requested to provide detailed information concerning the child’s food consumption patterns over three consecutive days (of which two were weekdays and one was weekend). Investigators instructed the parents on how to record food consumption and how to complete the dietary questionnaire. Collated food consumption records were then used to calculate calories and nutrients intakes. The latter were converted to percentages of the Recommended Dietary Allowance RDA or Dietary Reference Intake DRI for calories and nutrients based on age- and gender-normalized DRIs. Adequate intake for each dietary element was defined as greater or equal to 100% DRI, borderline intake was defined as 80–99% DRI, and inadequate intake was defined as less than 80% DRI. Detailed description of all food and beverages consumed, including cooking methods and the amount of each ingredient, was recorded. The conversion of household measures to grams was achieved through the use of pre-prepared list of commonly used household measures in Egypt.

Nutrient intake was calculated using the computer software program recommended by World Food Dietary Assessment System (WFDAS) [8]. The daily intakes of calories and protein were compared with National Research Council [9], while vitamins and minerals were compared with USDA, 2005 [10]. Each recruited child underwent complete physical examination; this included anthropometric measures according to the recommendations of the International Biological program [11]. The body height was measured to the nearest 0.1 cm on a Holtain portable anthropometer, and the body weight was determined to the nearest 0.01 Kg on Seca scale balance with the subject wearing minimal clothing and no shoes. Body mass index (BMI) was calculated as body weight (in kilograms) divided by body height (in meters) squared. Calculated BMI plotted by age on the Egyptian sex-specific growth chart [12]. Additionally, a socio-demographic and eating habits questionnaire was employed to capture information on participants’ age and date of birth, birth condition, eating habits, family information, life style and medical history.

### Statistical Methods

Statistical analysis was performed using the SPSS (Chicago, IL, USA) software for Windows (SPSS, 2010), data was expressed as Mean ±SE and t-test was used for numerical variables.

## Results

The participated children were all overweight: the BMI – z scores of group 1 were 0.95 ± 0.04 and 0.4 ± 0.3 and for group 2 were 0.9 ± 0.05 and 1.6 ± 0.5 for both sexes (boys and girls) respectively and showed high significant in Group 2. The cutoff point for the BMI- z score was ± 0.2 (Table 1).

**Table 1:** Mean ± SE of the anthropometric measures of study subjects (N=80)

Variable	Group 1		Group 2	
Weight (Kg)	21.15 ±1.14		37.17 ± 1.02	

	F	M	F	M

	20.22 ± 1.22	20.89 ± 1.33	33.10 ± 1.04	34.87 ± 1.03

Height (m)	108.32 ± 1.20		131.08 ± 0.87	

	F	M	F	M

	107.95 ± 2.21	109.65 ± 1.25	132.21 ± 1.02	133.06 ± 1.25

BMI	F	M	F	M

	18.89 ± 1.42	19.06 ± 2.57	19.02 ± 1.05	19.71 ± 1.70

BMI-z score	0.4 ± 0.3	0.95 ± 0.04	1.6 ± 0.5	0.9 ± 0.05

Results revealed that autistic children consumed average amount of calories: group 1 = 83% and group 2 = 94% of RDAs for their age. On the same hand, fat intake was more than the RDAs for their age with high saturated fats content (Table 2). Carbohydrates also were within average intake (54—60%) of the total caloric intake but shifted towards the upper-end of normal range for the older age group (6-9 years old). Protein intakes (expressed in grams) were slightly high when compared with the expected RDA but within normal range when calculated as a percentage (%) of total energy intake. Fibers and cholesterol intakes were within the average values of RDAs for age and sex.

**Table 2:** Mean ± SE of the main daily nutrients intake and the % of RDA of study subjects (N=80)

Variable	Group 1			Group 2		

	Intake	RDA		Intake	RDA	

	Mean ± SE	%	RDA	Mean ± SE	%	RDA
Energy (Kcal)	1490.98 ± 58.51	82.83	1800	1875.82 ± 55.32	93.79%	2000

Protein (g)	32.77 ± 3.69	8.79	24 g 10-30% of total energy	35.58 ± 7.95	7.12%	28 g 10-30 %of total energy

Total CHO (g)	202.23 ± 7.51	54.25	45-65 % of energy	297.79 ± 32.54	59.56%	45-65 % of energy

Fiber (g)	19.56 ± 2.33	69.85	23- 28 (girls) 25- 31 (boys)	23.57 ± 2.85	93.53%	23-28

Total Fat (g)	61.22 ± 4.61	36.95	39 - 62 (25 - 35% of calories)	60.26 ± 11.80	27.12%	62 - 85 (25 - 35% calories)

Saturated fat (g)	21.27 ± 1.45	13.09	16 to 18 (<10% calories)	29.63 ± 11.30	13.33%	< 20 girls: 18-22 boys:20-24 (<10% calories)

Mono Saturated fat (g)	17.25 ± 1.57	10.41	5-10 %of energy	23.61 ± 2.21	10.62%	5-10 %of energy

Poly Unsaturated fat (g)	9.35 ± 1.69	5.64	6-10% of energy	12.35 ± 1.02	5.56%	6-10% of energy

Cholesterol (mg)	289.19 ± 14.75	96.39	<300	258.03 ± 21.05	86.01%	<300

*p<0.005 versus RDA, **p<0.0001 versus RDA, ***p<0.00001 versus RDA.

It was noted that vitamin D intakes in both groups were below the RDAs for participants’ ages (group 1 = 46.8% and group 2 = 21.95%) and group2 showed highly significant P value < 0.01 compared to group 1 (P value < 0.05). Furthermore, vitamin C intakes were low ranged between borderline and inadequate intake in both groups with significant difference compared to their RDAs (88.64 % and 79.98 % for their ages, group 1 and group 2 respectively). Borderline intakes of folate, phosphorus, magnesium, and zinc were also noticed in the two age groups with significant differences compared to their RDAs. Group 2 showed low ranged levels of folate and zinc while Mg showed a significant difference in group 1 (p < 0.005). Low Calcium intake was also noted in the two groups and highly significant in group2 (p < 0.0001). Iron intake was inadequate in both groups: group 1 (p < 0.005) and group 2 (p < 0.0001) (Table 3). 

**Table 3:** Mean ±SE of the Vitamins & Minerals intake and the % of RDA of study subjects (N=80)

Variable	Group 1			Group 2		
	Intake	RDA		Intake	RDA	
	Mean ± SE	%	RDA /DRI	Mean ± SE	%	RDA /DRI
Vitamin A (µg RE)	560.19 ± 35.58	112.03	500	789.25 ± 32.58	108.32	700
Vitamin C (mg)	39.89 ± 5.41	88.64*	45	35.99 ± 3.09	79.98*	45
Vitamin D (mcg)	2.34 ± 0.68	46.80*	5	1.95 ± 0.37	21.95**	5
Vitamin E (TR)	5.99 ± 0.55	85.57	7	7.21 ± 1.01	92.03	7
Vitamin B6 (mg)	0.91 ± 0.49	151.66	0.6	1.51 ± 0.27	151.0	1
Vitamin B12 (mcg)	1.75 ± 1.26	145.83	1.2	2.29 ± 0.97	127.22	1.8
Thiamin (mg)	0.89 ± 0.60	148.33	0.6	1.40 ± 1.07	155.56	0.9
Riboflavin (mg)	0.88 ± 0.10	146.66	0.6	1.28 ± 0.14	142.22	0.9
Niacin (mg)	10.09 ± 5.23	126.12	8	13.96 ± 4.35	116.33	12
Folate (mg)	195.55 ± 27.21	97.63	200	231.03 ± 16.97	77.01*	300
Calcium (mg)	643.51 ± 37.21	80.04*	800	661.08 ± 49.21	50.89**	1300
Iron (mg)	8.01 ± 1.20	79.90*	10	6.99 ± 0.98	68.90**	10
Phosphorus (mg)	434.28 ± 35.11	86.86	500	1195.25 ± 68.96	95.62	1250
Magnesium (mg)	109.27 ± 18.20	84.05*	130	214.20 ± 18.23	89.25	240
Zinc (mg)	8.8 ± 6.75	88.60	10	8.86 ± 1.84	88.60*	10

* p<0.005 versus RDA

** p<0.0001 versus RDA

*** p<0.00001 versus RDA.

## Discussion

Despite the fact that feeding problems (e.g. food selectivity, avoidance behaviors and idiosyncratic) are prevalent complications among children with ASD; however this study revealed that they are prone to overweight and more vulnerable to associated negative consequences [13]. It is documented that autistic children are 1.42 times higher risk of developing obesity when compared to non-autistic children. The National Survey of Children’s Health revealed that prevalence of obesity among children with autism was 30.4% compared to 23.6% among children without autism [14]. In previous study to evaluate the nutritional status of children with special needs in Egypt, it was reported that incidence of obesity among autistic males and females were increased 15.8% and 16.1%, respectively [15]. Nevertheless, an elevated BMI z score among studied samples (overweight when plotted on the Egyptian sex-specific growth chart) was indicated for both age groups.

Unusual dietary patterns, limited access to physical activity and increased sedentary lifestyle could potentially contribute to overweight among children with ASD. Rosser and Frey, 2003 reported that opportunities to engage children with ASD in structured activities may be limited. This may further decline with age for children with ASD especially in developing countries [16].

In the present study, the adequacy of dietary intake of children with autism was assessed by comparing the mean dietary intake for each nutrient to the published RDA norms for calories, protein, carbohydrates and fats for individuals of same age group [9]. The data showed average mean values for calories, and carbohydrates however, much higher mean value for proteins and fat than the RDA was detected. Emond et al, 2010 [17] reported that children with autism spectrum demonstrated feeding problems and had a less varied diet, but energy intake and growth were not impaired, consistent with a previous study by Ronald et al. (2006) [17]. While Levy et al 2007 [18, 19] reported adequate intake for calories, carbohydrates, fat but increased for protein, Emond et al 2010 reported no differences in the balance of carbohydrates and proteins between children with ASDs and their peers.

Low intake of vitamin D and Calcium were observed in participated autistic children compared to RDAs for their ages and more obvious in older age group (6-9 years) [17]. Selective, with aversions to specific textures, colors, smells, and temperatures and rigidity with respect to specific brands of foods may become more obvious with age in addition to less control of parents eating habits. Bandini et al. (2010) [20] reported that children with ASDs would exhibit more food selectivity than typically developing children and that food selectivity would decline with age in typically developing children but would not be associated with age in children with ASD. Herndon et al.(2009) [21] compared the dietary intake of children with autistic-spectrum disorders to that of children with typical development, they noted that large proportions of children did not meet the national recommendations for daily intake of vitamin D and calcium. These data confirmed the study which had done by Meguid et al. (2010) [22] where they reported significant low 25(OH)D, 25(OH)D 1,25(OH)2D as well as lower calcium serum values in Egyptian children with ASDs than the controls. Furthermore, severe gestational vitamin D deficiency in rats produces pups with increased brain size and enlarged ventricles [23], anatomical abnormalities similar to those found in autism [24]. Maintaining vitamin D sufficiency in utero and during early life, to satisfy the vitamin D receptor transcriptional activity in the brain, may be important for brain development as well as for maintenance of mental function later in life [25].

Vitamin C has a reputation for its involvement in a plethora of metabolic, antioxidant, and bio-synthetic pathways, as a cofactor for certain enzymes necessary for neurotransmitter synthesis. On the other hand, Folic acid is essential to numerous metabolic pathways, several researchers reported favorable effects of folic acid on patients with autism [26].

While, the results of this study showed that vitamin C and folate contents were ranged between borderline and inadequate intake in both groups (group 1 and group 2) compared to their RDAs (p<0.005), however vitamin A was on the other hand adequate. This could be attributed to the consumption of fewer servings (below 5–10 servings /day) of fruits and vegetables of both age groups than what is currently recommended by the USDA [17]. Inadequate intake of folic acid and vitamin C has also been reported among autistic children in China [27].

On the positive side, nutrients intake showed an increase in vitamin B_6_ intake while vitamin E remained adequate this is in agreement with Xia et al.(2010) [27] who reported elevated vitamin B_6_ intake. In comparison, a recent smaller, detailed study that compared 3-day dietary diaries of children with ASD and children with typical development revealed that children with ASD consumed more vitamins B_6_ [11]. On the other hand, Cornish 1998 reported low intake of vitamin B_6_ in approximately half of participated children in his study, this could be due to ethnic dietary habit variations [28].

Although the path and etiology of autism is still poorly understood, some studies have reported that nutritional intervention can be of significant benefit to subjects with autism. A double-blind, placebo-controlled study reported that broad-based multivitamins and minerals supplementation was significantly helpful in reducing sleep and gastrointestinal symptoms in autistic children [27]. It has also been reported that oral supplementation with vitamin B_6_ significantly improved social interactions, communication, and intellectual function in children with autism [29]. Other studies have reported similar improvements in autistic children supplemented with vitamin B_6_, folate, and vitamin C [30].

There is considerable body of evidences about the important role of iron on cognitive, behavioral and motor development [31]. Iron is a component/coenzyme of many enzymes involved in neurotransmitter synthesis. In case of iron deficiency, due to decreased activity of associated enzymes, monoamine neurotransmitter systems may be affected [31]. High prevalence of iron deficiency has been reported in children with ASD [21] and inadequate dietary iron intakes were considered among the main cause of iron deficiency, and low iron intake was thought to be associated with food selectivity which is commonly seen in ASD children. Two large studies of iron status revealed that US and Canadian children with autism had anemia in 8% and 16% of cases respectively [32, 33]. The current study showed that iron intake was inadequate in the both groups: group 1 (p < 0.005) and group 2 (p < 0.0001).

Among its many functions, zinc is needed for the development and maintenance of the brain, adrenal glands, GI tract, and immune system [26]. Serotonin synthesis relies on zinc-activated enzymes; and zinc is also essential for antioxidant enzyme activity and other proteins important for growth and homeostasis [26]. Breeding experiments with rodents indicate a zinc deficiency in the mother can be passed on to the offspring and negatively influence immunity and brain development [34]. The current study showed insufficient intake of zinc especially in group 2, which is in agreement with recent study by Hyman et al. (2012) [35] on children with ASD aged 4 to 8 years who consumed significantly less Zinc in their diet.

The current study also reported that there was less concern from the mothers in terms of providing a balanced diet for their children when they got older. It has been identified from their history that some parents of older children were aware about gluten free, casein free diet and they applied it when feeding their children. This might be one of the reasons for some nutrient deficiency especially calcium, Mg and P (rich sources from dairy products, meat, wheat and nuts) [35].

In conclusion, children with autism should be regularly monitored for nutritional status and dietary intake. In addition, parents and guardians of autistic children should be provided with accurate nutritional information and should be encouraged to diversify the diet of their child or otherwise rectify the deficiencies, through appropriate supplementation with vitamins and minerals. Targeted, individualized nutritional therapy is crucial to managing the complexity of patients with ASD which could promote symptom amelioration and reduce co-morbidities in children with autism.
